# Association of Biosecurity and Hygiene Practices with Environmental Contamination with Influenza A Viruses in Live Bird Markets, Bangladesh

**DOI:** 10.3201/eid2609.191029

**Published:** 2020-09

**Authors:** Sukanta Chowdhury, Eduardo Azziz-Baumgartner, James C. Kile, Md. A. Hoque, Mohammed Z. Rahman, Md. E. Hossain, Probir K. Ghosh, Syed S.U. Ahmed, Erin D. Kennedy, Katharine Sturm-Ramirez, Emily S. Gurley

**Affiliations:** icddr,b, Dhaka, Bangladesh (S. Chowdhury, M.Z. Rahman, M.E. Hossain, P.K. Ghosh, S.S.U. Ahmed, K. Sturm-Ramirez, E.S. Gurley);; Chattogram Veterinary and Animal Sciences University, Chattogram, Bangladesh (S. Chowdhury, M.A. Hoque);; Centers for Disease Control and Prevention, Atlanta, Georgia, USA (E. Azziz-Baumgartner, J.C. Kile, E.D. Kennedy, K. Sturm-Ramirez);; Johns Hopkins Bloomberg School of Public Health, Baltimore, Maryland, USA (E.S. Gurley)

**Keywords:** influenza A virus, influenza virus, viruses, influenza, biosecurity, hygiene practices, environmental contamination, live bird markets, food safety, respiratory infections, zoonoses, Bangladesh

## Abstract

In Bangladesh, live bird market environments are frequently contaminated with avian influenza viruses. Shop-level biosecurity practices might increase risk for environmental contamination. We sought to determine which shop-level biosecurity practices were associated with environmental contamination. We surveyed 800 poultry shops to describe biosecurity practices and collect environmental samples. Samples from 205 (26%) shops were positive for influenza A viral RNA, 108 (14%) for H9, and 60 (8%) for H5. Shops that slaughtered poultry, kept poultry overnight, remained open without rest days, had uneven muddy floors, held poultry on the floor, and housed sick and healthy poultry together were more frequently positive for influenza A viruses. Reported monthly cleaning seemed protective, but disinfection practices were not otherwise associated with influenza A virus detection. Slaughtering, keeping poultry overnight, weekly rest days, infrastructure, and disinfection practices could be targets for interventions to reduce environmental contamination.

Highly pathogenic avian influenza A(H5N1) virus causes outbreaks in poultry and sporadic infections in humans globally (*1*,*2*). H5N1 virus is endemic to poultry in several countries in Southeast Asia, including Bangladesh, and causes major economic loss, as well as human illness and death (*1*,*3*–*5*). During 2007–2018, Bangladesh reported >550 highly pathogenic avian influenza outbreaks in poultry, 90% of which were reported from commercial poultry farms (*2*). Since 2008, eight human H5N1 cases, including 1 death, have been reported in Bangladesh; 3 of these cases were in live bird market (LBM) workers presumably exposed to infected poultry in the LBM (*1*). Vietnam, Thailand, Indonesia, Hong Kong, China, and Cambodia have also reported human cases of H5N1 infection with a history of poultry exposure in LBMs, suggesting that LBMs can facilitate spread of H5N1 infection among poultry and from poultry to humans (*6*,*7*).

Bangladesh has a large number of LBMs in urban areas in which multiple poultry species from backyard and commercial farms are housed together for sale; several studies detected highly pathogenic and low pathogenicity avian influenza viruses (AIVs) in LBM poultry and the environment (*8*–*13*). An LBM-based surveillance detected AIVs in waterfowl (4%) and environmental samples from poultry markets (29%). During 2007–2012, many subtypes, including H5N1, H5N2, H7N9, and H9N2, were identified in waterfowl and environmental samples (*14*). In 1 study, 9 (2%) of 450 LBM workers from 12 LBMs across Bangladesh had antibodies against H5N1 virus (*15*). Such findings suggest that environmental contamination with AIVs occurs in Bangladesh and that poultry workers are at risk for contracting AIVs from infected poultry in LBMs and their contaminated environment.

Affected countries have introduced interventions to reduce the spread of AIVs in LBMs, including temporary or permanent LBM closure, banning overnight poultry storage, and mandatory rest day(s), as well as daily cleaning of surfaces to reduce environmental contamination (*16*–*22*). Temporary, weekly 1-day closures at live poultry markets in Guangzhou, China, was implemented for effective disinfection in response to the H7N9 outbreaks during 2013–2014 (*23*). However, market-level interventions have not been effective in reducing environmental contamination in Bangladesh. The infrastructure and daily activities of individual poultry shops within markets are heterogeneous (*9*). Because individual poultry shops have their own infrastructure and biosecurity controls, shop-level analyses might be useful in developing and designing effective interventions. Our study aimed to assess the shop-level prevalence of influenza A virus contamination among LBM shops across Bangladesh and to identify biosecurity and hygiene practices that are associated with risk for and protection from influenza A virus contamination.

## Methods

**Figure 1:**
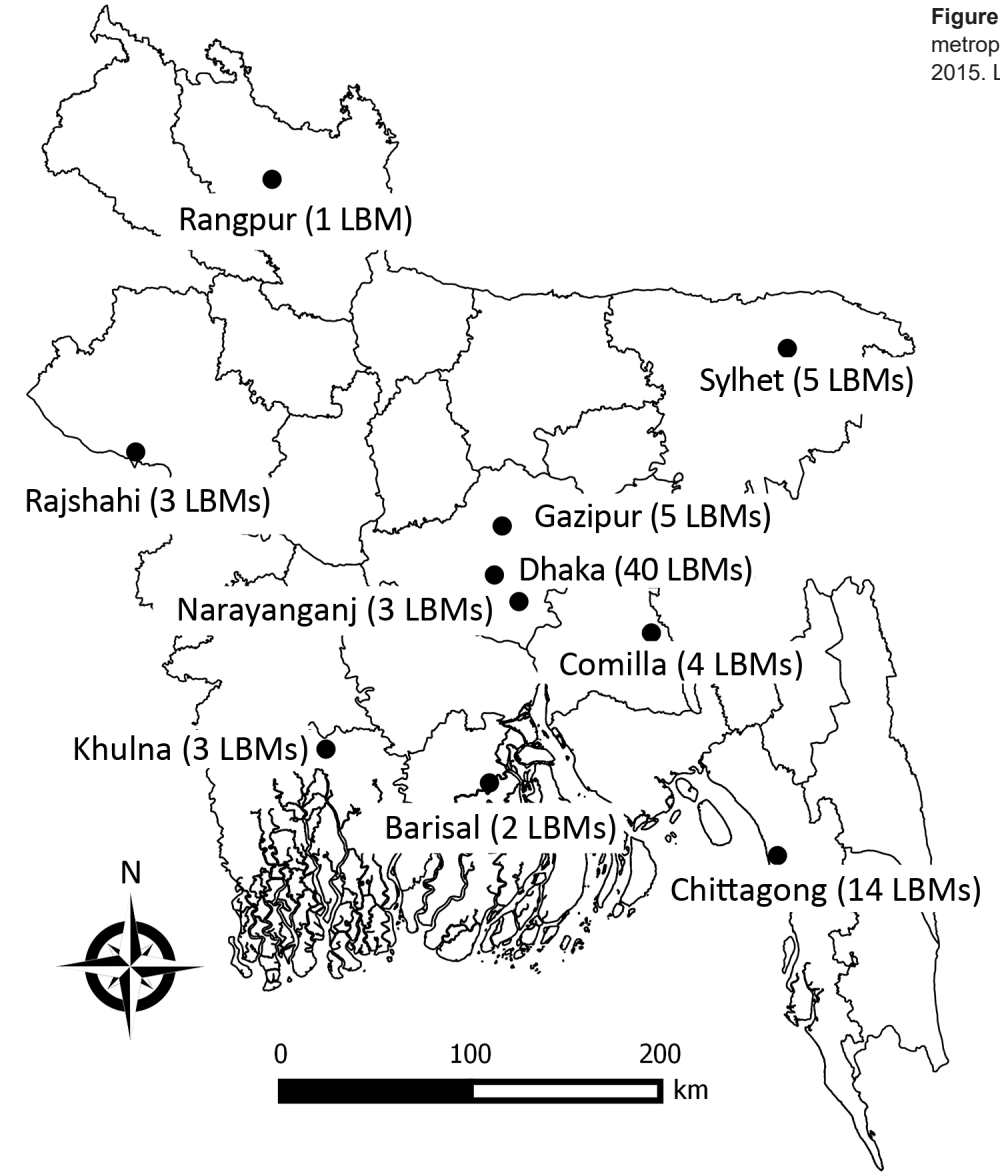
Locations of LBMs, 10 metropolitan areas, Bangladesh, March 2015. LBM, live bird market.

Bangladesh has 10 metropolitan areas where large numbers of LBMs are located. We conducted a cross-sectional study in all 10 areas (Figure 1).

### Selection of LBMs and Poultry Shops

We determined that we needed 800 poultry shops to detect >1% prevalence of AIV with 95% confidence and 0.7% precision. Initially, the field team visited each metropolitan area to identify all LBMs and count the number of individually owned poultry shops in each market. After visiting all the cities, we prepared a list of LBMs with >10 poultry shops for each metropolitan area. We then selected 80 LBMs from 10 metropolitan areas by using a proportionate random sampling technique. Finally, we enrolled 10 shops in each LBM by using a random number generator.

### Biosecurity Measures and Other Practices

During March 2015, the field team visited each selected shop to interview poultry shop owners or workers and collect information about shop characteristics, poultry transactions, and biosecurity and hygiene practices. On the basis of previously identified risk factors and recommended biosecurity and hygiene practices, we hypothesized that cleaning, disinfection, overnight poultry storage, a weekly rest day, practice of poultry slaughtering within shops, type of floor, poultry holding areas, presence of waterfowl, poultry density, number of poultry species, source of poultry, and the separation of sick poultry from healthy poultry could be associated with the detection of AIV in the poultry shop environment (Appendix 1) (*11*,*17*,*19*,*21*,*22*,*24*–*28*). In a questionnaire (Appendix 2), we defined cleaning as “cleaning of poultry holding areas with water and/or broom,” and we defined disinfection as “cleaning of poultry holding areas with a disinfectant.” We asked owners whether they cleaned poultry holding areas daily, weekly, monthly, or did not clean within the past month. We asked whether they disinfected poultry holding areas weekly, monthly, or did not disinfect within the last month. The field team also collected some market-level information by interviewing members of the market committee.

### Sample Collection

From each selected shop, we collected 8–10 swab specimens of poultry droppings, cages, feed, drinking water, slaughtering surfaces and utensils, slaughtering by-products, offal, shop floors, or waste bins. We pooled the 8–10 samples from each shop and tested them as a single sample. Some shops had no slaughtering facilities within their premises. From these shops, we collected swab specimens from other sources, including poultry droppings, cages, feed, and drinking water. We collected 1 pooled sample from each of 800 selected shops during March 2015 because highly pathogenic avian influenza (H5N1) activity typically peaks during January–March (*29*).

### Laboratory Testing

We used a real-time reverse transcription PCR detection kit for typing and subtyping influenza viruses and fluorescent TaqMan probes at the icddr,b (*30*). Primers and probes specific for the matrix gene were used to detect influenza A viruses. To identify H5, H7, and H9 subtypes in influenza A virus–positive samples, we used H5, H7, and H9 hemagglutinin gene–specific primers and probes (*30*).

### Observations

On the basis of laboratory testing results, we identified all influenza A/H5–positive shops and an equal number of influenza A virus–negative shops by using a random number generator and a list of influenza A virus–negative shops. Field staff observed each selected shop for a 3-hour period during April 2015. Staff observed cleaning and disinfection activities of selected poultry shops during surprise visits at times when cleaning activities were scheduled. Field staffs were blinded to the laboratory test results of selected shops.

### Statistical Analysis

**Figure 2:**
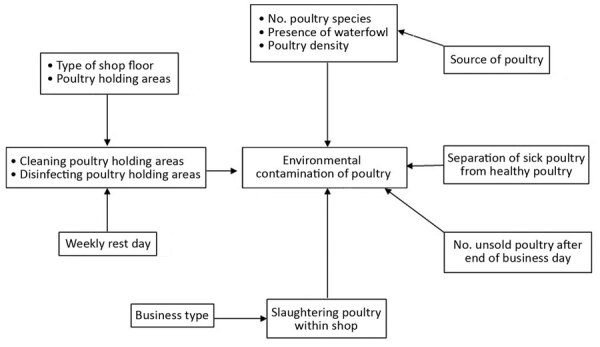
Conceptual framework for shop-level environmental contamination with avian influenza viruses in live bird markets, Bangladesh, March 2015.

We summarized characteristics of poultry shops, including infrastructure and biosecurity and hygiene measures, by using descriptive analyses. We estimated the presence of environmental contamination with influenza A viruses in shops and 95% CIs. Initially, we constructed a conceptual framework to identify causal association and confounders as described (*31*) (Figure 2). We then performed univariate analyses to estimate odds ratios (ORs). Exposure variables associated with outcomes with p <0.2 in univariate analysis and confounder variables from the conceptual framework were selected for multivariate analyses. We used backward stepwise selection of variables with a significance level of 0.05 to construct models. We then used mixed-effect logistic regression multivariate models, accounting for clustering by metropolitan area and market, to estimate adjusted ORs (aORs). We assessed collinearity by calculating the variance inflation factor for independent variables used in the regression models (*32*). Weekly cleaning was highly correlated with daily cleaning practices; therefore, we removed weekly cleaning from the model during multivariate analyses. We calculated model χ^2^ and R^2^ (the coefficient of determination) to measure goodness-of-fit for multivariate regression model. We performed all statistical analyses by using Stata version 13 software (StataCorp LLC, https://www.stata.com).

### Ethics

Field staff obtained written consent from shop owners or poultry workers for data and sample collection from their shops. The icddr,b Research Review Committee and Ethical Review Committee reviewed and approved the study protocol (protocol no. PR-15012).

## Results

We identified 104 LBMs that had ≥10 poultry shops. Among these LBMs, we selected 800 shops in 80 LBMs for sample and data collection (Table 1). The average number of poultry shops in each market was 20 (SD 10.5, range 10–55). Most (77%) poultry shops were retail and sold live poultry directly to consumers. The average size of each poultry shop was 9 m^2^, and the average duration of trade per day was 14 hours. Chickens were the predominant poultry species sold at LBMs, and 91% of shops had >1 poultry species the day of our visit (Table 2). A total of 6% of shops sold waterfowl only, and 4% sold chickens and ducks.

**Table 1:** Influenza A and avian influenza virus contamination of live bird market shops, by metropolitan areas, Bangladesh, March 2015

Metropolitan cities	No. live bird markets investigated	Total no. shops tested	No. (%) shops positive for influenza A	No. (%) shops positive for influenza A/H5	No. (%) shops positive for influenza A/H9
Dhaka	40	400	116 (29)	46 (12)	52 (13)
Chittagong	14	140	15 (12)	3(2)	7 (5)
Gazipur	5	50	14 (28)	1 (2)	9 (18)
Sylhet	5	50	25 (50)	2 (4)	21 (42)
Comilla	4	40	5 (13)	0	3 (8)
Rajshahi	3	30	7 (23)	1 (3)	4 (13)
Khulna	3	30	3 (10)	0	2 (7)
Narayanganj	3	30	10 (33)	4 (13)	5 (17)
Barisal	2	20	5 (25)	0	3 (15)
Rangpur	1	10	5 (50)	3 (30)	2 (20)
Total	80	800	205 (26)	60 (8)	108 (14)

**Table 2:** Daily poultry trade at 800 live bird market shops selected for the study, by poultry species, Bangladesh, March 2015

Poultry sold	No. (%) shops	Mean no. poultry/day (range)		
		Stocked/day	Sold/day	Leftover/day
Only chicken	722 (90)	210 (20–3,760)	159 (10–3,760)	52 (0–1,650)
Only waterfowl	3 (1)	130 (50–290)	108 (32–252)	22 (10–38)
Only pigeon	5 (1)	90 (50–150)	41 (5–80)	49 (30–70)
Two poultry species	57 (7)	267 (20–1333)	185 (10–850)	83 (5–895)
More than 2 poultry species	13 (2)	522 (48–3,000)	296 (33–1,075)	227 (10–1,925)

Poultry shopkeepers housed poultry in different types of settings, including wire cages, bamboo cages, and on the floor. Most (80%) poultry shops had uneven floor surfaces, partially made with tiles/concrete and mud. Poultry shop owners collected poultry from different sources, including wholesale markets, intermediaries, and directly from poultry farms. Most (86%) poultry shops slaughtered poultry on premises. Cleaning and disinfecting practices varied among poultry shops: 468 shops (59%) reported cleaning poultry holding areas daily, 185 (23%) reported using a disinfectant once a week, 592 (74%) reported frequently working throughout the week (i.e., not following the recommended weekly day of rest), and 654 shops (82%) reported keeping unsold poultry after the end of each business day.

### Laboratory Results for Environmental Specimens

Environmental specimens from 205 (26%, 95% CI 23%–29%) shops were positive for influenza A viral RNA; 108 (14%, 95% CI 11%–16%) were positive for the H9 subtype and 60 (8%, 95% CI 6%–9%) were positive for the H5 subtype (Table 1). An additional 37 (5%, 95% CI 3%–6%) influenza A–positive shops had samples that were not subtypeable with H5, H7, and H9 primers. Samples from 29 (4%) shops were confirmed for both H5 and H9 subtypes. No samples were positive for H7 (95% CI 0%–0.5%). Shops in all 10 cities had at >1 sample positive for influenza A viral RNA, and 7 cities (70%) had shops positive for the H5 subtype. Among the 80 LBMs, >1 shop from 74 markets (93%) was positive for influenza A viral RNA, and >1 shop from 35 markets (44%) was positive for influenza A/H5 RNA. Environmental samples from 6 LBMs (3 from Chittagong, 1 from Dhaka, 1 from Khulna, and 1 from Comilla) were negative for influenza A viral RNA.

### Observational Findings

We conducted observations in 60 influenza A/H5 virus–positive and 60 influenza A virus–negative shops. We did not find any major differences in cleaning and disinfection practices between influenza A/H5 virus–positive and influenza A virus–negative shops. Surveyors observed cleanings in 85% of influenza A/H5 virus–positive shops and 86% of influenza A virus–negative shops. Among these shops, only 2% of influenza A/H5 virus–positive shops performed disinfection by using washing powder or another recognized disinfectant, whereas 3% of influenza A virus–negative shops performed disinfection during our period of observation.

### Associations between Shop-Level Biosecurity, Hygiene, and AIV Environmental Surface Contamination with Influenza A Viruses

We showed by using univariate analyses that poultry shops that kept poultry on the floor (OR 3.86, 95% C: 1–15.07; p = 0.05), slaughtered poultry within the shop (OR 1.7, 95% CI 1.08–2.67; p = 0.02), had unsold poultry after the end of the business day (OR 2.29, 95% CI 1.44–3.63; p<0.01), did not rest 1 day a week (OR 1.34, 95% CI 1.14–1.58; p = 0.01), kept sick and healthy appearing poultry together (OR 1.25, 95% CI 1–1.58; p = 0.05), and had uneven floor surfaces (partly made with tiles/concrete and mud) (OR 4.01, 95% CI 2.53–6.36; p<0.01) were more likely to be positive for influenza A viral RNA in environmental samples compared with shops that did not have these characteristics (Table 3). Poultry shops that reportedly cleaned poultry holding areas either daily (OR 0.41, 95% CI 0.27–0.62; p<0.01), weekly (OR 0.37, 95% CI 0.18–0.73; p<0.01), or monthly (OR 0.2, 95% CI 0.08–0.49; p<0.01), and had weekly disinfection (OR 0.81, 95% CI 0.61–1.07; p = 0.14) seemed less likely to be positive for influenza A viral RNA compared with shops that did not.

**Table 3:** Shop-level biosecurity practices and environmental contamination with 800 influenza A viruses in 10 metropolitan areas, Bangladesh, March 2015*

Variable	No. (%) shops	No. (%) shops positive for influenza A viruses, n = 205	OR (95% CI)	p value	Adjusted OR (95% CI)†	p value†
Poultry species						
Single	731 (91)	184 (25)	Referent	NA		
Multiple	69 (9)	21 (30)	1.45 (0.9–2.32)	0.12		
Presence of waterfowl						
No	752 (94)	190 (25)	Referent	NA		
Yes	48 (6)	15 (31)	1.68 (0.86–3.32)	0.13		
Poultry holding areas						
Only wire cage	281 (35)	55 (20)	Referent	NA	Referent	NA
Only bamboo cage	153 (19)	53 (35)	2.12 (0.85–5.28)	0.1	2.24 (0.87–5.77)	0.09
Only floor	24 (3)	9 (38)	3.86 (1–15.07)	0.05	3.95 (1.27–12.23)	0.01
Mixed	342 (43)	88 (26)	1.72 (0.96–3.09)	0.06	1.71 (0.96–3.04)	0.06
Cleaning poultry holding areas						
No cleaning in past month	26 (3)	12 (46)	Referent	NA	Referent	NA
Monthly	68 (9)	10 (14)	0.2 (0.08–0.49)	<0.01	0.47 (0.28–0.8)	<0.01
Weekly‡	238 (30)	57 (24)	0.37 (0.18–0.73)	<0.01	NA	NA
Daily	468 (59)	126 (27)	0.41 (0.27–0.62)	<0.01	1.09 (0.91–1.31)	0.31
Disinfecting poultry holding areas						
No disinfection in past month	577 (72)	150 (26)	Referent	NA		
Monthly	38 (5)	10 (26)	1.1 (0.53–2.25)	0.79		
Weekly	185 (23)	45 (24)	0.81 (0.61–1.07)	0.14		
Slaughtering poultry within shop						
No	115 (14)	18 (16)	Referent	NA	Referent	NA
Yes	685 (86)	187 (27)	1.7 (1.08–2.67)	0.02	1.87 (1.11–3.14)	0.01
Presence of unsold poultry after the end of business day						
No poultry left	146 (18)	19 (13)	Referent	NA	Referent	NA
Presence of unsold poultry	654 (82)	186 (28)	2.29 (1.44–3.63)	<0.01	2.35 (1.4–3.93)	<0.01
Weekly rest day						
Yes	208 (26)	51 (25)	Referent	NA	Referent	NA
No	592 (74)	154 (26)	1.34 (1.14–1.58)	<0.01	1.35 (1.12–1.63)	<0.01
Source of poultry						
Poultry farm	49 (6)	12 (24)	Referent	NA		
Intermediaries	54 (7)	10 (19)	0.85 (0.27–2.64)	0.78		
Wholesale market	525 (66)	143 (27)	1.05 (0.51–2.16)	0.88		
Multiple sources	172 (21)	40 (23)	0.94 (0.35–2.49)	0.9		
Separation of sick poultry from healthy flocks						
Yes	357 (45)	85 (24)	Referent	NA	Referent	NA
No	443 (55)	120 (27)	1.25 (1–1.58)	0.05	1.31 (1.06–1.62)	0.01
Type of shop floor						
Tiles/concrete	244 (31)	32 (13)	Referent	NA	Referent	NA
Dirt/mud	33 (4)	8 (24)	3.61 (1.7–7.67)	<0.01	3.2 (1.46–7.09)	<0.01
Mixed	523 (65)	165 (32)	4.01 (2.53–6.36)	<0.01	3.64 (2.32–5.71)	<0.01
Poultry density/mm^2^						
<32	568 (71)	147 (26)	Referent	NA		
>33	232 (29)	58 (25)	0.89 (0.68–1.15)	0.39		

*Variables for geographic location of metropolitan areas and live bird markets were adjusted to account for clustering effects in univariate and multivariate analysis. NA, not applicable; OR, odds ratio. †Only statistically significant relationships are shown for adjusted OR (95% CI) data and corresponding p values. ‡The weekly cleaning variable was removed from the multivariate model because of collinearity. Model fit: model χ^2^ 76.29, p<0.001, df 11; adjusted generalized R^2^ 0.596.

In the final multivariate analysis model, we showed that poultry shops that slaughtered poultry within the shop (aOR 1.87, 95% CI 1.11–3.14; p = 0.01), had unsold poultry after the end of the business day (aOR 2.35, 95% CI 1.4–3.93; p<0.01), did not rest 1 day a week (aOR 1.35, 95% CI 1.12–1.63; p<0.01), had uneven floor surfaces (partly made with tiles/concrete and mud) (aOR 3.64, 95% CI 2.32–5.71; p<0.01), held poultry on the floor (aOR 3.95, 95% CI 1.27–12.23; p = 0.01), and kept sick and healthy appearing poultry together (aOR 1.31, 95% CI 1.06–1.62; p = 0.01) were significantly more likely to be positive for influenza A viruses compared with shops that did not report these characteristics (Table 3). Reported monthly cleaning was protective (aOR 0.47, 95% CI 0.28–0.8; p<0.01), but disinfecting practices of poultry holding areas was still not significantly associated with influenza A virus detection in the multivariate model (p = 0.85). The final model selected seemed to fit data well (χ^2^ 76.29, df 11, p<0.001, and R^2^ 0.596). No market-level factors, including central cleaning and disinfection practices, were significantly associated with influenza A virus detection in the multivariate model (Appendix 1).

## Discussion

Evaluation of existing biosecurity and hygiene practices is necessary to develop and design interventions to reduce the spread of AIVs in LBMs. Our study provides a detailed depiction of the daily operation of poultry shops and current biosecurity and hygiene practices in selected LBMs of Bangladesh. We identified certain biosecurity and hygiene practices associated with environmental contamination with AIVs: slaughtering poultry within shops, having unsold poultry after the end of the business day, skipping rest days, uneven floor surfaces, holding poultry on the floor, and keeping sick and healthy appearing poultry together.

Our study determined that most shops did not implement biosecurity practices, which have reduced AIV in other countries. For example, biosecurity and hygiene practices, including weekly rest days, depopulation, and cleaning with disinfectant, reduced the risk for AIV detection in poultry and environmental specimens in China (*28*). The prevalence of H7N9 virus in environmental specimens from LBMs in China decreased after the closure of live poultry markets (*33*). Daily waste removal was found to be protective in Indonesia (*17*). In the United States, environmental contamination decreased after implementing routine cleaning and disinfection (*19*,*22*). Although monthly cleaning was found to be protective in reducing environmental contamination with AIVs in this study, most shops in Bangladesh do not disinfect, and their current biosecurity practices do not seem to prevent environmental contamination. Moreover, most of the studied shops had rough dirt and mud floors that are less suitable for proper cleaning and disinfection, indicating poor market infrastructure.

Globally, countries reporting human cases of AIV also have LBMs contaminated with AIVs. AIV contamination of LBM environments increases the risk for infection and amplification of the virus in virus-free birds. In addition, if the AIV is zoonotic, as are H7N9, H5N1, and H5N6 viruses, increased viremia in birds increases the risk for human exposure and infection. For example, in Vietnam, AIVs were detected in 3.2% of poultry specimens collected from LBMs; in Egypt, H5N1 virus was detected in poultry in 12.4% of LBMs; in China, H7N9 virus was detected in 10% of environmental specimens from LBMs; in Indonesia, AIVs were detected in 47% of environmental specimens from LBMs; in Thailand, H5N1 virus was detected in 3.1% of market poultry; and in Bangladesh, AIVs were detected in 23% of poultry specimens (*10*,*17*,*33*–*35*). In our study, >90% of the LBMs were positive for influenza A viruses, and 44% were specifically positive for AIV H5 RNA. Detection of AIV RNA in environmental samples indicates that market poultry were infected with AIVs near the time of sample collection and might excrete, secrete, or contaminate surfaces and humans through their carcasses, feathers, and offal. Our study findings also confirmed the presence of 2 subtypes (H5 and H9) of AIV, which might lead to genetic reassortment and evolution of new AIV strains in poultry of public health concern (*29*).

Epidemiologic studies have described the effectiveness of weekly or monthly rest days in reducing environmental contamination of LBMs with AIV (*21*,*24*). The number of human cases of infection with H7N9 virus has been observed to be reduced after permanent or temporary closure of LBMs and the culling of poultry (*24*,*25*,*33*,*36*). The government of Bangladesh imposed an order in 2012 to practice weekly rest days for cleaning and disinfecting LBMs within Dhaka (*37*). Nevertheless, 1 study found 74% of poultry shop owners did not practice weekly rest days, which might increase the risk for environmental contamination. A weekly rest day should be enforced by the government to decrease the risk for AIV circulation in LBMs.

Unsold poultry can play a major role in maintaining virus circulation in markets (*25*). Unsold infected poultry can infect incoming poultry, promoting further transmission of influenza viruses in susceptible birds. Banning overnight poultry storage in China reduced H9N2 virus isolation in chickens (84%) (*24*). In our study, most (82%) poultry shops reported that they stored poultry overnight in their shops to sell the next day. A previous study from Bangladesh also found that 73% of poultry shops kept poultry in their stalls for >1 day (*38*).

Slaughtering by-products, such as blood and offal, of AIV-infected poultry provide the most likely opportunity for environmental contamination and subsequent human exposure to high loads of virus. In Indonesia, slaughtering poultry within market premises was a risk factor for environmental contamination (*17*,*26*). H7N9 virus was detected in swab samples collected from surfaces of chopping boards in China (*33*). Persons from China and Bangladesh prefer to purchase live chickens that are slaughtered in the market at the time of purchase (*9*,*36*). A study suggested introducing central slaughtering of all live poultry in the LBM to control the risk posed of AIVs (*39*). In Bangladesh, most poultry shops, including those in this study, sold and slaughtered poultry within their shop (*9*). This practice might increase the risk for AIV contamination and perpetuate the exposure of poultry to AIV in LBMs. Although our study did not assess AIV transmission within LBMs, we cannot rule out the risk for AIV transmission to humans through slaughtering of infected poultry. We recommend introducing centralized slaughter facilities in LBMs to decrease the spread of AIV.

LBMs in Bangladesh are larger (ranging from 10 to 55 poultry shops) than those in Hong Kong, where the number of poultry shops in each LBM was 3–24 (*21*). Maintaining effective biosecurity and hygiene measures might be more difficult in larger LBMs that had poor infrastructure. The infrastructures of LBMs in city areas were quite similar. However, the prevalence of H5 and H9 subtypes varied between cities and might naturally differ in virus ecology by farm or geographic site. The infrastructure of our studied poultry shops within LBMs was often rudimentary: most were fully enclosed by walls, but most had rough muddy floors, unsystematic poultry holding areas, poor waste disposal systems, and unconfined slaughtering facilities. Urban markets have more poultry shops than rural markets. Urban LBMs usually are open every day, whereas rural LBMs are open once or twice per week. Bangladesh should consider investing in poultry shop infrastructure improvements and biosecurity practices, particularly in city areas, to better control environmental contamination with AIVs.

In China, poultry trading networks linked with LBMs were strongly associated with a higher prevalence of H7N9 virus among poultry and risk for H7N9 transmission to humans (*36*). Movement of infected poultry between markets has a major role in the spread of AIVs from 1 market to another (*17*,*40*). Poultry market supply chains in urban areas of Bangladesh are complex, collecting poultry from different sources, including directly from farms, intermediaries, or wholesale markets. These complex networks might promote a high number of contacts between infected and susceptible marketed birds and, therefore, increase AIV transmission potential within the trade networks.

This cross-sectional study design might have limited interpretation of some of the results. Although AIV circulation and amplification at LBMs are continuous processes influenced by time-dependent parameters, such as time to last cleaning before sampling and time to last poultry introduction/mixing before sampling, we only examined environmental contamination for AIVs at 1 point in time and did not explore time from last cleaning or disinfection. No additional laboratory tests were performed to characterize viral load and viability of AIVs detected because of limited funding. Therefore, it is unclear if the AIVs detected during the study were infectious to humans. The information we collected from poultry shop owners and workers about biosecurity might have been affected by social desirability bias, which might have underestimated the prevalence of practices that place shop at risk for contamination with AIVs.

In conclusion, our study identified risky practices, hygiene, and infrastructure in Bangladesh LBMs associated with an increased likelihood of shop contamination with AIVs. Improvement of these biosecurity practices, such as removing poultry at the end of the day, observing weekly rest days, introducing centralized slaughter facilities, and regular cleaning and disinfection, might help to prevent AIV contamination. LBM infrastructure, including floors, poultry holding areas, waste disposal systems, and slaughtering facilities, also need improvement. Potential valuable shop-level interventions to address these deficiencies in biosecurity practices might include training for poultry shop owners and poultry workers about effective biosecurity practices to reduce AIV contamination and the risk AIV poses to humans in Bangladesh.

Appendix 1 Additional information on association of biosecurity and hygiene practices with environmental contamination with influenza A viruses in live bird markets, Bangladesh.

Appendix 2 Questionnaires used for study of association of biosecurity and hygiene practices with environmental contamination with influenza A viruses in live bird markets, Bangladesh.
