# Supplementary material for: Association of Biosecurity and Hygiene Practices with Environmental Contamination with Influenza A Viruses in Live Bird Markets, Bangladesh
Source: Emerg Infect Dis. 2020 Sep;26(9):2087–96. doi: 10.3201/eid2609.191029 (PMC7454050; doi:10.3201/eid2609.191029)
Supplement: Appendix 1 — Additional information on association of biosecurity and hygiene practices with environmental contamination with influenza A viruses in live bird markets, Bangladesh. [file 19-1029-Techapp-s1.pdf]

Article DOI: https://doi.org/10.3201/eid2609.191029

Association between Biosecurity and Hygiene Practices with Environmental Contamination with Influenza A Viruses in Live Bird Markets, Bangladesh

Appendix 1

Appendix 1 Table 1. Description of biosecurity practices and their potential association with environmental contamination for avian influenza viruses

Biosecurity practices	Categories	Role in AIV epidemiology and environmental contamination
Cleaning poultry holding areas	Not cleaning	Retain environmental waste that increase the risk for environmental contamination
	Monthly	Retain environmental waste that increase the risk for environmental contamination
	Daily	Remove environmental waste that decrease the risk for environmental contamination
Disinfecting poultry holding areas	Not disinfecting	Retain virus within environmental premises that increase the risk for environmental contamination
	Monthly	Retain virus within environmental premises that increase the risk for environmental contamination
	Weekly	Reduce environmental contamination by killing virus
Weekly rest day	Yes	Supportive for cleaning and disinfection
	No	Not supportive for cleaning and disinfection
Number of unsold poultry after the end of business day	No poultry left	Prevent amplification of avian influenza viruses
	Presence of unsold poultry	Maintain and amplify avian influenza viruses
Slaughtering poultry within shop	Yes	Increase environmental contamination
	No	Decrease environmental contamination
Separation of sick poultry from healthy flock	Yes	Limits physical contact between birds
	No	Increase risk for avian influenza virus transmission

Appendix 1 Table 2. Market-level biosecurity practices and environmental contamination with 80 influenza A viruses in 10 metropolitan cities, Bangladesh, March 2015*

Variable	No. LBMs positive for influenza A viruses, n = 74	No. LBMs negative for influenza A viruses, n = 6	Prevalence ratio (95% CI)
Poultry density/mm^2			
≤32 poultry	49 (66)	5 (83)	Referent
≥33 poultry	25 (34)	1 (17)	1.05 (0.92–1.21)
Cleaning managed by market committee			
No	26 (35)	2 (33)	Referent
Yes	48 (65)	4 (67)	0.99 (0.91–1.08)
Disinfection managed by market committee			
No	49 (66)	4 (67)	Referent
Yes	25 (34)	2 (33)	1 (0.84–1.18)
Presence of drain for liquid waste disposal			
Present	56 (76)	4 (67)	Referent
Absent	18 (24)	2 (33)	0.98 (0.95–1.01)
Presence of central slaughtering facility			
Yes	16 (22)	1 (17)	Referent
No	58 (78)	5 (83)	0.97 (0.84–1.13)

*Values are no. (%). LBM, live bird markets.
