# Supplementary material for: Association of Biosecurity and Hygiene Practices with Environmental Contamination with Influenza A Viruses in Live Bird Markets, Bangladesh
Source: Emerg Infect Dis. 2020 Sep;26(9):2087–96. doi: 10.3201/eid2609.191029 (PMC7454050; doi:10.3201/eid2609.191029)
Supplement: Appendix 2 — Questionnaires used for study of association of biosecurity and hygiene practices with environmental contamination with influenza A viruses in live bird markets, Bangladesh. [file 19-1029-Techapp-s2.pdf]

Article DOI: https://doi.org/10.3201/eid2609.19.1029

Association of Biosecurity and Hygiene Practices with Environmental Contamination with Influenza A Viruses in Live Bird Markets, Bangladesh

Appendix 2

Appendix 2 Table 1. Questionnaire for LBM shop data about cleaning, disinfection, infrastructure and biosecurity practices

1.	Market name:			Shop ID:	
	Address:			Date:	
	Interviewer's name:				
2.	Name of the interviewee:				
3.	Profession (√):<br>• Shop owner<br>• poultry worker<br>• middlemen<br>• others………………………				
4.	Number of employee:				
5.	Type of poultry business (√):	• Retail<br>• wholesale<br>• mixed			
6.	Average number of active poultry transactions day per month:				Day
7.	Average length of poultry selling time/day		Starting time	End time	
8.	Poultry holding area (√)<br>• Cages<br>• floor<br>• bamboo nest<br>• Other...				
9.	Number and type of poultry being kept for sale per day:				
	Poultry type		Average number of poultry are kept/day	Average number of poultry are sold/day	Average number of leftover poultry after a business day
	Chickens	Broilers			
		Layer			
		Breeders			
		Backyard			
		Sonali			
	Waterfowl	Ducks			
		Geese			
	Pigeons				
	Quails				
	Others………………...				
10.	How many leftover poultry remain today ?			Health status of leftover poultry	
	Species (√)	Number		Health status (√)	Number
	• Chicken			• Sick	
	• Duck			• Dead	
	• Geese			• Healthy	
	• Pigeon				
	• Quail				
11.	Last 2 d poultry morbidity (numbers)	Chicken			
		Duck			
		Geese			
		Pigeon			
		Quail			

1.	Market name:		Shop ID:	
12.	Last 2 d poultry morbidity (numbers)	Chicken		
		Duck		
		Geese		
		Pigeon		
		Quail		

13.	Source of poultry (√):	• Wholesale market<br>• Commercial farms<br>• Backyard farms<br>• Multiple sources (market and farm)<br>• Middlemen<br>• others

14.	What types of activities carried in your shop (√)?						
	Activities	Chicken	Duck	Geese	Quail	Pigeon	Others
	Selling						
	Slaughtering						
	Hot water dressing						
	Manual defeathering						
	Evisceration						
	cutting meat						
	Others						

15.	Poultry arrival time at market:	(√)	(%)
	In morning (5 a.m.-11.59 a.m.)		
	At noon (12 p.m.-12.59 p.m.		
	In afternoon (1 p.m.- 5.59 p.m.)		
	In evening (6 p.m.-7.59 p.m.)		
	At night (8 p.m.-11.59 p.m.)		
	Late at night (12 a.m.-4:59 a.m.)		

16.	Management of leftover poultry at the end of daily selling (√):
	• Keeping for sale on next day<br>• Supply to other shop<br>• Supply to restaurant<br>• Others

17.	Disposal of slaughter waste (√):	Solid waste	Liquid waste
	Through drain		
	In drum		
	In dust bin		
	In the open space		
	Others:		
	No disposal		
18.	Do you separate sick poultry from healthy poultry (√)?	• Yes<br>• No	

19.	Management of sick poultry (√):
	• Slaughter for sale<br>• Wait for recovery<br>• Sale sick poultry<br>• Others.............................
20.	Outcome of slaughtered sick poultry (√):
	• Sell to the regular customer<br>• Sell to the regular restaurant<br>• Slaughter for self consumption<br>• Slaughter for destruction<br>• Others
21.	Management of dead poultry (√):
	• Disposed through drain<br>• Disposed in drum<br>• Disposed in dust bin<br>• Disposed in the open space<br>• Others:

22.	Condition of waste bin (drum)		• Porous<br>• sealed				
23.	Shop cleaning (√):		• Wet cleaning<br>• Dry cleaning<br>• Others................				
	Wet clean	Place/Materials	Using clean water/	Daily basis/ weekly basis	How many	How many	How many

1.	Market name:	Shop ID:	

			drain water/ reuse water		times/ day	times/ Week	times/ month
		Floor					
		Work surface (bench/chopping board)					
		Utensils					
		Cages and other poultry holding area					
		Others..................					
	Dry clean	Place/Materials	sweeping/using shovel/ brushing/grooming	Daily basis/ weekly basis	How many times/ day	How many times/ Week	How many times/ month
		Floor					
		Work surface (bench/chopping board)					
		Utensils					
		Cages and other poultry holding area					
		Others..................					

24.	Shop disinfecting (√): Yes / No						
	Place/ Materials	Daily basis/weekly basis	How many times/ day	How many times/week	How many times/month	Type of disinfectant (Virkon S/Timsen/Emsen/ bleac hing powder/deter gent/soap/ other......)	Provided by (self/employee/ market committee/City Corporation/N GO/FAO/other........)
	Floor						
	Work surface (bench/choppin g board)						
	Utensils						
	Cages and other poultry holding area						
	Others						

25.	Placement of poultry during cleaning and disinfection	Same cage/other cage/ other place.................................

26.	Water and others used for (√):	cleaning waste	dressed chicken	hand
	Supply water			
	Store water			
	Recycling contaminated water			
	Drain water			
	No water supply			
	Using towels, paper (others....................)			

27.	Floor of shop (√)	Yes	No	Comments
	Tiles			Full/partial
	Concreted			Full/partial
	Dirt			Full/partial
	Others.................			

28.	Construction of floor	Smooth/rough/mixed/others.........................	
29.	Shop size (for poultry density)	Floor length	Floor width

30.	Location of shop (√)
	• Adjacent to other poultry shop<br>• Not adjacent to other poultry shop
31.	Have you visited other poultry shop of this market today? • Yes • No
32.	Have you visited poultry shop of other market today? • Yes • No

1.	Market name:	Shop ID:	
33.	Do your shop has machine hot water dressing	• Yes<br>• No	
	If Yes, what percentage of poultry dress daily using machine		(%)
34.	When cleaning and disinfection performed more frequently of a day<br>• 8 a.m. –11 a.m. (morning)<br>• 11 a.m. - 2 p.m. (noon)<br>• 2 p.m. - 5 p.m. (after noon)<br>• 5 p.m. - 8 p.m. (evening)<br>• 8 p.m. –11 p.m. (night)<br>• Others..		
35.	Other relevant information		

Appendix 2 Table 2. Data sheet for collecting data about cleaning, disinfection, infrastructure and biosecurity practices from poultry shop by observation method

Market ID: Market name:	
Shop ID:<br>Date of Observation:<br>Observation time: AM/PM to AM/PM	
**Infrastructure of LBM and shop**	
1) Floor type **(√)**:<br>• concreted<br>• tiled<br>• muddy<br>• partial concrete partial muddy<br>• others.......................................<br>2) Floor surface **(√)**:<br>• Smooth<br>• rough<br>• others..<br>3) Drainage system **(√)**:<br>• No drain	
• active drain<br>• drain with stagnant waste<br>• others..................................	
4) Poultry holding area **(√)**:<br>• Cages<br>• floor<br>• bamboo nest<br>• others...	
**Cleaning and disinfection practices**	
1) Cleaning **(√)**	• No cleaning<br>• Wet cleaning (e.g., using water)<br>• Dry cleaning (e.g., using groom or brush)<br>• Mixed (both wet and dry)<br>• Other...
2) Type of water supply **(√):**	• Supply water<br>• Store water<br>• Recycling contaminated water<br>• Drain water<br>• No water supply<br>• Using towels and paper<br>• others..
3) Disinfection **(√)**	• Yes<br>• No
4) Appearance of disinfectant in shop **(√)**	• Vircon S<br>• Detergent<br>• Bleaching powder<br>• Soapy water<br>• Others..
5) Disinfection performed by (during observation) **(√)**	• Shop owner<br>• Poultry workers<br>• Government workers<br>• NGO workers<br>• City corporation workers<br>• Market (employed by committee) workers

Market ID: Market name:	
	• f) others..
6) Management of poultry during cleaning and disinfection **(√)**:<br>• keeping at same cage<br>• keeping in other cage<br>• keeping in isolated house<br>• others..	
**Biosecurity practices**	
1) Disposal of dead poultry **(√)**	• Within disposal drum<br>• within polythene bag<br>• on the floor<br>• within cage<br>• purchase<br>• central dust bin
	• others.....................................

2) Disposal of slaughtering waste **(√)**	Solid waste	Liquid waste
	• Within disposal drum<br>• within polythene bag<br>• on the floor<br>• within cage<br>• purchase<br>• central dust bin<br>• others................................	• Within disposal drum<br>• within polythene bag<br>• on the floor<br>• within cage<br>• purchase<br>• central dust bin<br>• others.....................................
3) Slaughtering site **(√)**	• Within same shop<br>• outside shop<br>• within an isolated area<br>• central slaughtering area<br>• other..	

4) Disposal of waste (feces/bedding material) from live poultry **(√)**	• Within disposal drum<br>• within polythene bag<br>• on the floor<br>• within cage<br>• purchase<br>• central dust bin<br>• others.....................................
5) Condition of waste bin /disposal drum	• Sealed<br>• porous<br>• Others............
6) Keeping multiple poultry species within same shop **(√)**	• Yes<br>• No
7) Keeping multiple types (breeder/layer/broiler) of poultry within same shop **(√)**	• Yes<br>• No
8) Keeping backyard and commercial (breeder/layer/broiler) poultry within same shop **(√)**	• Yes<br>• No
9) Keeping duck/geese (breeder/layer/broiler) with chicken within same shop **(√)**	• Yes<br>• No

10) Poultry and poultry holder statistics of the shop

Type of holding area	Number	Types of poultry	Total number
Cages		Chicken	
Bamboo nests		Duck	
Floor		Geese	
Others...		Quail	
		Pigeon	
		Others................................	

11) Processing slaughtered poultry **(√)**	• Manual defeathering<br>• Manual evisceration<br>• Cutting meat<br>• Boiled water dressing<br>• Others..
12) Presence of boiled water dressing machine **(√)**	• Yes<br>• No
Others	

Appendix 2 Table 3. Questionnaire for collecting market level data from member of market committee

Market ID			Date:
1	Market name:		
2	Name of interviewer and designation:		
3	Name of interviewee and designation:		
4	Number of total shops:		
5	Type of market (√):<br>• Wholesale<br>• retail<br>• mixed		
6	Average poultry transaction time per day:	Hours (start) to Hours (end)	
7	Opening schedule of poultry market (√):	• Everyday<br>• 2 d/week<br>• 5 d/week<br>• 6 d/week<br>• Others......................................	
8	Number of days/week market is closed:	Number | Name of the Day	
9	Market cleaning by committee (√)	• Daily<br>• Every alternative day<br>• Weekly<br>• Monthly<br>• No cleaning<br>• Others	
10	Frequency of cleaning (√)	• once a day<br>• two times/day<br>• three times/day<br>• once/week<br>• twice/week<br>• others..	
11	Cleaning by (√):<br>• water<br>• sweeping<br>• Brush<br>• Broom<br>• others……………………………………………………		
12	**Who clean market ? (√)**<br>• workers recruited by committee<br>• City Corporation workers<br>• Shop workers<br>• Others..		
13	**Places where cleaning is performed (√)**<br>• Central walkway<br>• Market floor<br>• individual shop floor<br>• drain<br>• central slaughtering place<br>• poultry carrying vehicles<br>• poultry holding areas (cages)<br>• wall<br>• Utensils (feeder, drinker)<br>• others..		
14	**Market disinfecting by committee (√):**	• Daily<br>• Every alternative day<br>• Weekly<br>• Monthly<br>• No disinfection<br>• Others......................................	
15	**Frequency of disinfection (√)**	• once/day<br>• two times/day<br>• three times/day<br>• once/week<br>• twice/week<br>• others..	
16	**Any specific day (used for disinfection) (√)**<br>• Yes	Name of the day	

	• No	
17	**Who perform disinfection ? (√)**<br>• workers recruited by committee<br>• City Corporation workers<br>• DLS (Department of Livestock Services) workers<br>• Shop workers<br>• NGO workers<br>• FAO (Food and Agriculture Organization) workers<br>• Others..	
18	**Places where disinfection is performed (√)**<br>• Central walkway<br>• Market floor<br>• individual shop floor<br>• drain<br>• central slaughtering place<br>• poultry carrying vehicles<br>• poultry holding areas (cages)<br>• wall	
	• others..	
19	**Disinfectants name (√)**<br>• Vircon S<br>• Timsen<br>• Emsen<br>• Bleaching powder<br>• Detergent<br>• Soapy water<br>• others..	
20	**Disinfectant source (√)**<br>• Purchase from local pharmacy/shop<br>• FAO<br>• NGO<br>• DLS<br>• City Corporation<br>• Government<br>• others..	
21	**Central waste management** (√)	
	Performed by (√)	Frequency (√)
	• workers recruited by committee<br>• City Corporation workers<br>• DLS workers<br>• Poultry shop workers<br>• NGO workers<br>• FAO workers<br>• Others..	• once/day<br>• two times/day<br>• three times/day<br>• once/week<br>• twice/week<br>• others..
22	**Drainage system (√)**<br>• No drain<br>• active drain<br>• drain with stagnant waste<br>• others..................................	
23	**Presence of central slaughtering place (√)**<br>• Yes<br>• No<br>• others..	
24	**Presence of isolated room for sick poultry (√)**<br>• Yes<br>• No	
25	**Presence of central dustbin for waste disposal (√)**<br>• Yes<br>• No	
